# Associations of household income and parental education with early childhood caries: the Japan Environment and Children’s Study

**DOI:** 10.1265/ehpm.25-00358

**Published:** 2026-03-26

**Authors:** Fumie Kaneko, Eri Yamada, Junji Miyazaki, Satoyo Ikehara, Ryo Kawasaki, Hiroyasu Iso, the Japan Environment and Children’s Study (JECS) Group

**Affiliations:** 1 Division of Public Health, Department of Social Medicine, The University of Osaka Graduate School of Medicine, Suita, Osaka, Japan; 2 Osaka Regional Center for Japan Environment and Children’s Study, The University of Osaka, Suita, Osaka, Japan; 3 Department of Epidemiology and Behavioral Science, Faculty of Medicine, University of the Ryukyus, Ginowan, Okinawa, Japan; 4 Institute for Global Health Policy Research, Bureau of Global Health Cooperation, Japan Institute for Health Security, Shinjuku, Tokyo, Japan

**Keywords:** Early childhood caries, Oral health, Socioeconomic status, Preschool children, Japan

## Abstract

**Background:**

Although the prevalence of early childhood caries (ECC) has declined over the past few decades, it remains a significant public health concern in Japan. Socioeconomic disparities in ECC risk have been reported; however, the extent to which these disparities exist across different indicators of socioeconomic status (SES) and the degree to which early oral health practices help explain them remain unclear. This study evaluated the association between parental SES and ECC and examined the extent to which early oral health behaviors contributed to these associations.

**Methods:**

We analyzed the data from 68,312 children and their parents participating in the Japan Environment and Children’s Study, a nationwide birth cohort recruited between 2011 and 2014. Parental SES, including household income equivalized using the OECD-modified equivalence scale and educational attainment, was assessed using self-reported questionnaires. Diagnoses of dental caries from birth to age 4 years were reported retrospectively by caregivers when the child was 4 years old. Multivariable logistic regression was used to examine associations between SES and ECC. Mediation analyses were conducted to evaluate whether oral health behaviors at age 2, including fluoride application, tooth brushing practices, continued bottle feeding, and frequency of between-meal snacks, mediated these associations. All models were adjusted for demographic and potential confounding factors.

**Results:**

Overall, 23.0% of children had been diagnosed with caries by age 4 years. The higher prevalence was consistently observed among children from lower SES backgrounds starting at around age 2 years. Both lower household income and lower parental educational attainment were associated with higher odds of ECC. The highest odds were observed among children whose parents had secondary-level education (adjusted odds ratio: 1.52; 95% confidence interval: 1.44–1.59). Although oral health behaviors mediated these associations, each behavior accounted for less than 5% of the total effect.

**Conclusion:**

Lower household income and parental educational attainment were consistently associated with a higher risk of ECC from early childhood. Mediation analyses using oral health behaviors assessed at age 2 indicated that these behaviors explained a small proportion of the observed socioeconomic disparities, suggesting a small contribution of individual behaviors to early-life socioeconomic inequalities in ECC.

**Supplementary information:**

The online version contains supplementary material available at https://doi.org/10.1265/ehpm.25-00358.

## Background

Early childhood caries (ECC) is one of the most prevalent chronic conditions in childhood, affecting a substantial proportion of children worldwide [1]. The prevalence of caries at ages 1.5 and 3 years has steadily declined over the past decades in Japan [2]. Dental caries is a multifactorial and dynamic disease influenced by biological, behavioral, and psychosocial factors shaped by the child’s environment [3]. It impairs daily functioning and quality of life, not only for affected children but also for their families, placing financial burdens and causing psychological stress for parents [4, 5].

Socioeconomic disparities in ECC have been documented both globally and in Japan [6–8]. A previous study of Japanese children born in 2001 suggested that socioeconomic disparities are already evident by age 2.5 years and tend to widen as they grow older [8]. Socioeconomic conditions have a notable influence during early childhood, when caregivers shape oral health practices [6]. However, the extent to which individually modifiable oral health behaviors contribute to socioeconomic disparities in ECC remains unclear.

Socioeconomic status (SES) is a multidimensional construct encompassing material resources as well as social and cognitive aspects [6, 7]. Household income and parental educational attainment are commonly used indicators of parental SES, as income generally reflects access to material living conditions, while education captures parental knowledge and skills that may influence health-related behaviors.

Using data from a large, nationwide birth cohort in Japan, this study had two main objectives. The first was to investigate the association between parental socioeconomic status (SES), measured by household income and educational attainment, and the cumulative incidence of dental caries by the age of 4 years. Second, we examined whether and to what extent individual-level oral health behaviors at 2 years of age, such as fluoride application, tooth brushing practices, continued bottle feeding, and the frequency of between-meal snacks, mediated the association between SES and the incidence of ECC between 3 and 4 years of age.

## Methods

### Study population

The present study was based on data obtained from the Japan Environment and Children’s Study (JECS), an ongoing nationwide prospective birth cohort study. The detailed protocol and baseline profile have been described previously [9, 10]. Briefly, women were recruited during early pregnancy at obstetric clinics, hospitals, or municipal offices across 15 Regional Centres between January 2011 and March 2014. Enrollment and follow-ups for mothers were conducted during the first, second/third trimesters, as well as at 1 and 6 months post-delivery. Follow-ups for offspring were simultaneously conducted every 6 months after childbirth, with the information on dental caries available in the age 4 questionnaire.

This study comprised a primary analysis assessing the association between parental SES and dental caries, and a secondary analysis evaluating the mediating effect of oral health behaviors on these associations. Among the 104,043 fetal records, we included 100,122 live births and conducted the primary analysis, excluding participants who did not respond to the age 4 questionnaire (n = 22,075), as well as those with missing data on caries diagnosis (n = 3,826), on any of the three socioeconomic variables: household income, paternal educational attainment, or maternal educational attainment (n = 5,467), and on the number of household members (n = 442). Furthermore, for the secondary analyses, we excluded children who had been diagnosed with caries before age 2 years (n = 5,577) to examine the extent to which oral health behaviors mediate the associations of household income and parental educational attainment with the risk of childhood dental caries. Consequently, 68,312 and 62,735 children were included in the primary and secondary analyses, respectively (Supplemental Fig. 1).

### Measurement

The exposure of interest was parental SES, including equivalized household income and parental educational attainment. All SES-related information was obtained from self-report questionnaires completed by mothers during the second trimester, whereas information on household composition was collected during the first trimester. Annual household income was originally collected using nine categorical options (in million yen): <2, 2–<4, 4–<6, 6–<8, 8–<10, 10–<12, 12–<15, 15–<20, and ≥20. These categories were recategorized by assigning a representative value (in million yen) to each category as follows: 2, 3, 5, 7, 9, 11, 13.5, 17.5, and 20, respectively. The resulting values were equivalized using the OECD-modified equivalence scale, which assigned a weight of 1.0 to the first adult in the household, 0.5 to each additional adult, and 0.3 to each child [11], and then categorized into quartiles. Paternal and maternal educational attainment were categorized into three groups: university or higher, vocational school or junior college, and high school or lower. Based on this information, we constructed a combined variable representing parental educational attainment, classified into four groups: both parents had post-secondary education; father had post-secondary and mother had secondary education; father had secondary, and mother had post-secondary education; and both parents had secondary education. Secondary education was defined as high school or lower, and post-secondary education was defined as vocational school, junior college, university, or higher, according to previous studies conducted in Japan [8].

The outcome of interest was the diagnoses of caries between birth and age 4. Information on children’s caries diagnoses was obtained from the questionnaire when the children were 4 years old. First, guardians were asked whether their children had ever been diagnosed with caries from birth to 4 years of age. If they answered “yes,” they were asked to select all applicable ages at which the diagnosis occurred (ages 0, 1, 2, 3, and 4). For the primary analyses, the outcome was defined as whether the child had ever been diagnosed with caries from birth to 4 years of age (yes/no). For the secondary analyses, the outcome was restricted to diagnoses that occurred between the ages of 3 and 4 to clarify the temporal relationship between exposure, outcome, and mediators.

Oral health behaviors, which served as mediators in the secondary analyses, were assessed using a questionnaire administered when the children were 2 years old. This questionnaire included information on fluoride application between birth and age 2, daily tooth brushing frequency, final brushing (i.e., parental assistance in removing residual plaque), toothpaste use, continued bottle feeding at age 2, and the frequency of between-meal snacks per day. Demographic information, including marital status and residential area, was obtained using a maternal questionnaire administered during the first trimester. Maternal age at delivery, child’s sex, and birth weight were extracted and transcribed from medical records. Household smoking status was assessed at 1 month of age. Information on the mother’s working hours was collected when the child was 1 year of age. Data on co-residence with grandparents were collected at 1.5 years of age, information on daycare attendance was obtained at 2 years of age, and the presence of siblings was confirmed at 2.5 years of age.

### Statistical analysis

For the primary analysis, general characteristics were summarized as frequencies and percentages. Second, we demonstrated the age-specific distribution of cumulative caries experience from ages 0 to 4 years, based on the number of distinct years in which dental caries was diagnosed. Children were classified into three groups: once (diagnosed in one year), twice (in two years), and three or more times (in three or more years). This classification reflects the number of years in which a diagnosis occurred, rather than the number of decayed teeth. Distributions were separately presented according to household income and parental educational attainment. Third, we assessed the association between SES factors and dental caries using multivariable logistic regression models conducted in two steps. First, we examined each SES factor independently: household income, paternal education, maternal education, and the combination of parental education. Model 1 adjusted for demographics and potential confounders identified in previous studies [12, 13]. In the second step, Model 2 was constructed and adjusted for other SES variables. Specifically, when household income was included, both paternal and maternal education levels were included as covariates. When paternal education was the exposure variable, household income and maternal education were adjusted for, and vice versa for maternal education. In the model that assessed the combination of parental education, only household income was included as a covariate. As a sensitivity analysis, we additionally restricted the analysis to children with married mothers.

For the secondary analyses, the distribution of oral health behaviors at age 2 and dental caries diagnoses between the ages of 3 and 4 years was first described across SES groups. To ensure appropriate temporal ordering of the exposure, mediator, and outcome, participants with caries diagnoses before the age of 2 were excluded from the secondary analysis. Household income was dichotomized at 2.77 million yen, which was the median equivalized household income in this study. Parental educational attainment was dichotomized into two categories: both parents with secondary education (i.e., high school or lower) and at least one parent with post-secondary education (i.e., vocational school, junior college, university, or higher). Each oral health behavior was dichotomized according to whether it followed the recommended practice specified in existing guidelines [14, 15], with the recommended category serving as the reference group. Participants with missing data for a given oral health behavior were excluded from the corresponding model. Multivariable logistic regression models were conducted to estimate adjusted odds ratios of each oral health behavior according to SES indicators. Mediation analyses were conducted using the PROC CAUSALMED procedure in SAS to evaluate the extent to which each oral health behavior mediated the association between SES indicators and dental caries. The confidence intervals for the natural direct and indirect effects were estimated using 1,000 bootstrap resamples. All mediation models included the same covariates as those used in the primary analysis. All statistical analyses were performed using SAS version 9.4 (SAS Institute Inc., Cary, NC, USA). Statistical significance was set at *p* < 0.05.

### Ethical consideration

The JECS protocol was reviewed and approved by the Ministry of the Environment’s Institutional Review Board on Epidemiological Studies and the Ethics Committees of all participating institutions. Written informed consent was obtained from all participants. The JECS study was conducted in accordance with the Declaration of Helsinki and other domestic regulations and guidelines in Japan.

## Results

### General characteristics

Among the 68,312 participants, 51.1% were boys (Table 1). The mean maternal age at delivery was 31.6 (standard deviation: 4.8), with 96.6% of mothers being married. Approximately 70% of mothers and 60% of fathers had attained post-secondary education. Mothers from higher-income families tended to be older at delivery, have higher educational attainment, and have partners with higher education. Nearly half of the children attended daycare at age 2, with higher attendance among those from the highest- and lowest-income families. Approximately 65% of the children had older and/or younger siblings at age 2, and nearly 20% lived with grandparents. In the highest-income households, 62.2% had no smokers, whereas in lowest-income households, 60.6% had at least one smoker, and 3.6% reported smoking in the presence of a child.

**Table 1:** General characteristics of the participants included for the primary analysis.

**Characteristics**	**All**	**Equivalized household income**							

		**Q1, lowest** **(<1.67 million yen)**		**Q2** **(1.67–<2.78 million yen)**		**Q3** **(2.78–<3.33 million yen)**		**Q4, highest** **(≥3.33 million yen)**	

		**Number (%)**		**Number (%)**		**Number (%)**		**Number (%)**	
Total participants	68312	18438	(27.0)	15483	(22.7)	18444	(27.0)	15947	(23.3)
Sex									
Boy	34929	9442	(51.2)	7903	(51.0)	9492	(51.5)	8092	(50.7)
Girl	33383	8996	(48.8)	7580	(49.0)	8952	(48.5)	7855	(49.3)
Birth weight, g									
<2,500	6070	1533	(8.3)	1444	(9.3)	1618	(8.8)	1475	(9.3)
≥2,500	62208	16900	(91.7)	14030	(90.6)	16816	(91.2)	14462	(90.7)
Missing	34	5	(0.0)	9	(0.1)	10	(0.1)	10	(0.1)
Maternal age at delivery, years									
<30	23627	8134	(44.1)	6141	(39.7)	5714	(31.0)	3638	(22.8)
30–34	25205	6281	(34.1)	5291	(34.2)	7261	(39.4)	6372	(40.0)
35–39	16206	3414	(18.5)	3391	(21.9)	4601	(25.0)	4800	(30.1)
≥40	3274	609	(3.3)	660	(4.3)	868	(4.7)	1137	(7.1)
Paternal educational attainment									
University or more	24859	3589	(19.5)	4560	(29.5)	7716	(41.8)	8994	(56.4)
Vocational school or junior college	15692	4345	(23.6)	3850	(24.9)	4323	(23.4)	3174	(19.9)
High school or less	27761	10504	(57.0)	7073	(45.7)	6405	(34.7)	3779	(23.7)
Maternal educational attainment									
University or more	16847	2139	(11.6)	2733	(17.7)	5019	(27.2)	6956	(43.6)
Vocational school or junior college	29744	7359	(39.9)	6968	(45.0)	8821	(47.8)	6596	(41.4)
High school or less	21721	8940	(48.5)	5782	(37.3)	4604	(25.0)	2395	(15.0)
Marital status									
Married	66009	17406	(94.4)	14840	(95.9)	18141	(98.4)	15622	(98.0)
Divorced, widowed, unmarried	2150	965	(5.2)	593	(3.8)	279	(1.5)	313	(2.0)
Missing	153	67	(0.4)	50	(0.3)	24	(0.1)	12	(0.1)
Mother’s working hours per week, hours									
Not working	44431	11047	(59.9)	10245	(66.2)	12616	(68.4)	10523	(66.0)
<20	4614	1787	(9.7)	1131	(7.3)	1062	(5.8)	634	(4.0)
20–<40	9339	3122	(16.9)	1953	(12.6)	2207	(12.0)	2057	(12.9)
≥40	6880	1556	(8.4)	1457	(9.4)	1846	(10.0)	2021	(12.7)
Missing	3048	926	(5.0)	697	(4.5)	713	(3.9)	712	(4.5)
Daycare attendance									
Attending	32822	8785	(47.7)	6578	(42.5)	8226	(44.6)	9233	(57.9)
Not attending	33288	8899	(48.3)	8442	(54.5)	9675	(52.5)	6272	(39.3)
Missing	2202	754	(4.1)	463	(3.0)	543	(2.9)	442	(2.8)
Having siblings at age two									
Both older and younger siblings	3922	1712	(9.3)	584	(3.8)	927	(5.0)	699	(4.4)
Only older siblings	32530	12047	(65.3)	5417	(35.0)	8550	(46.4)	6516	(40.9)
Only younger siblings	8539	1203	(6.5)	2652	(17.1)	2406	(13.0)	2278	(14.3)
No siblings	21490	2826	(15.3)	6419	(41.5)	6147	(33.3)	6098	(38.2)
Missing	1831	650	(3.5)	411	(2.7)	414	(2.2)	356	(2.2)
Living with grandparents									
Yes	13058	5324	(28.9)	3963	(25.6)	1844	(10.0)	1927	(12.1)
No	53806	12599	(68.3)	11215	(72.4)	16268	(88.2)	13724	(86.1)
Missing	1448	515	(2.8)	305	(2.0)	332	(1.8)	296	(1.9)
Household smoking									
No smoker	34546	7125	(38.6)	7322	(47.3)	10182	(55.2)	9917	(62.2)
Not near children	31933	10518	(57.1)	7742	(50.0)	7901	(42.8)	5772	(36.2)
Near children	1402	663	(3.6)	320	(2.1)	255	(1.4)	164	(1.0)
Missing	431	132	(0.7)	99	(0.6)	106	(0.6)	94	(0.6)
Residential area									
Hokkaido	5497	1327	(7.2)	1208	(7.8)	1654	(9.0)	1308	(8.2)
Miyagi	5661	2195	(11.9)	1478	(9.6)	1132	(6.1)	856	(5.4)
Fukushima	8036	2483	(13.5)	2037	(13.2)	1953	(10.6)	1563	(9.8)
Chiba	3976	959	(5.2)	908	(5.9)	1117	(6.1)	992	(6.2)
Kanagawa	4428	714	(3.9)	851	(5.5)	1348	(7.3)	1515	(9.5)
Koshin	4887	1352	(7.3)	1149	(7.4)	1248	(6.8)	1138	(7.1)
Toyama	3967	809	(4.4)	890	(5.8)	1134	(6.2)	1134	(7.1)
Aichi	3929	763	(4.1)	803	(5.2)	1230	(6.7)	1133	(7.1)
Kyoto	2833	594	(3.2)	570	(3.7)	806	(4.4)	863	(5.4)
Osaka	5636	1610	(8.7)	1305	(8.4)	1572	(8.5)	1149	(7.2)
Hyogo	3637	743	(4.0)	741	(4.8)	1135	(6.2)	1018	(6.4)
Tottori	2122	627	(3.4)	513	(3.3)	537	(2.9)	445	(2.8)
Kochi	4641	1390	(7.5)	974	(6.3)	1237	(6.7)	1040	(6.5)
Fukuoka	5436	1210	(6.6)	1207	(7.8)	1628	(8.8)	1391	(8.7)
South Kyusyu and Okinawa	3626	1662	(9.0)	849	(5.5)	713	(3.9)	402	(2.5)

### Associations between SES and dental caries

**Fig. 1:**
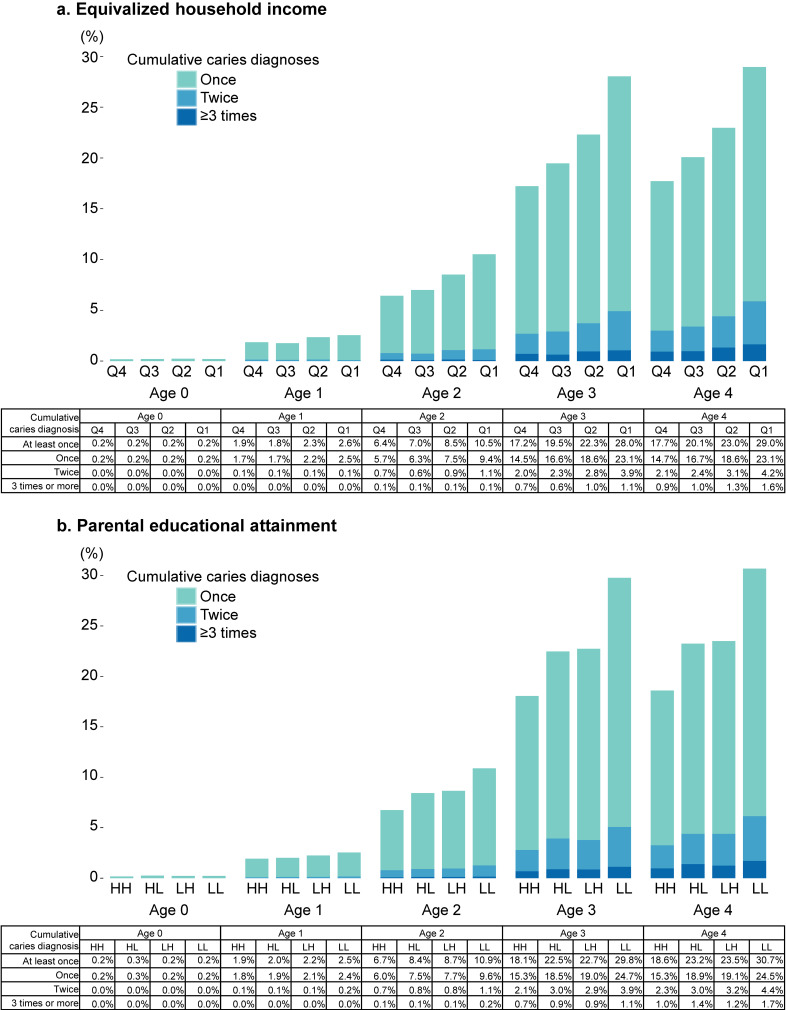
Age-specific cumulative caries diagnoses according to socioeconomic factors. (a) Equivalized household income, (b) Parental educational attainment. Equivalized household income was categorized into quartiles: Q4 (≥3.33 million yen), Q3 (2.78–<3.33 million yen), Q2 (1.67–<2.78 million yen), and Q1 (<1.67 million yen). Parental education was categorized into four groups: HH (both parents had postsecondary education), HL (father had postsecondary education and mother had secondary education), LH (father had secondary education and mother had postsecondary education), and LL (both parents had secondary education). H (postsecondary education) refers to vocational school, junior college, university, or higher education’ L (secondary education) refers to high school or less. Note: Cumulative caries diagnoses represent the number of distinct years (ages 0–4) in which the child was diagnosed with dental caries, based on guardian reports collected at age 4. Children were classified into three groups: once (diagnosed in one year), twice (in two years), and three or more times (in three or more years).

Overall, 23.0% of the children had experienced a dental caries diagnosis by age 4 years; inverse associations between SES and the cumulative prevalence of dental caries became apparent from age 2 years onward (Fig. 1a). At age 4 years, the proportion of children with at least one dental caries diagnosis was 18.0%, 20.4%, 23.4%, and 29.5% across households for the highest to lowest income quartiles, respectively. Similarly, the cumulative prevalence of caries was notably higher among children whose parents had a secondary-level education (Fig. 1b). In addition to the cumulative prevalence of having at least one caries diagnosis, a socioeconomic gradient was also observed when classifying children by the number of years in which they were diagnosed. At age 4, the proportions of children who had been diagnosed in one, two, or three or more years were consistently lower among those from higher-income households and those with parents who had higher educational attainment.

Household income, paternal and maternal educational attainment, and the combination of parental educational attainment were independently associated with dental caries (Table 2). In Model 2, which was additionally adjusted for other SES factors, children from households in the lowest income quartile had higher odds of caries than those from households in the highest income quartile; the adjusted odds ratio (OR) and 95% confidence interval (CI) were 1.24 (1.17–1.32). When paternal and maternal educational levels were examined separately, lower educational attainment for each parent was associated with higher odds of caries, with a progressive increase across the educational attainment groups. Compared to paternal university or higher education, the ORs (95% CIs) of caries were 1.08 (1.02–1.14) for vocational school or junior college, and 1.25 (1.19–1.31) for high school or less. A similar increasing trend was observed for maternal education. This association was more pronounced when the combination of parental educational attainment was incorporated. The group in which both parents had secondary education showed the highest odds of caries, with an adjusted OR of 1.52 (1.44–1.59), compared with the group in which both parents completed post-secondary education. The results did not change materially in a sensitivity analysis restricted to children with married mothers (Supplementary Table 1).

**Table 2:** Odds ratios and 95% confidence intervals of early childhood caries according to socioeconomic factors.

**Socioeconomic factors**	**No. of participants**	**No. (%) of case**	**Crude OR** **(95% CI)**	**Model 1** **Adjusted OR^a^** **(95% CI)**	**Model 2** **Adjusted OR^b^** **(95% CI)**
**Equivalized household income**					
Q4, highest (≥3.33 million yen)	15947	2869 (18.0)	1.00 (Reference)	1.00 (Reference)	1.00 (Reference)
Q3 (2.78–<3.33 million yen)	18444	3756 (20.4)	1.17 (1.10–1.23)	1.01 (1.04–1.16)	1.05 (0.99–1.11)
Q2 (1.67–<2.78 million yen)	15483	3619 (23.4)	1.39 (1.32–1.47)	1.27 (1.20–1.34)	1.16 (1.09–1.23)
Q1, lowest (<1.67 million yen)	18438	5446 (29.5)	1.91 (1.82–2.01)	1.40 (1.33–1.48)	1.24 (1.17–1.32)
**Paternal educational attainment**					
University or higher	24859	4583 (18.4)	1.00 (Reference)	1.00 (Reference)	1.00 (Reference)
Vocational school or junior college	15692	3456 (22.0)	1.25 (1.19–1.31)	1.14 (1.08–1.20)	1.08 (1.02–1.14)
High school or less	27761	7651 (27.6)	1.68 (1.62–1.75)	1.40 (1.34–1.47)	1.25 (1.19–1.31)
**Maternal educational attainment**					
University or higher	16847	3026 (18.0)	1.00 (Reference)	1.00 (Reference)	1.00 (Reference)
Vocational school or junior college	29744	6469 (21.8)	1.27 (1.21–1.33)	1.14 (1.08–1.19)	1.05 (1.00–1.11)
High school or less	21721	6195 (28.5)	1.82 (1.74–1.91)	1.49 (1.41–1.57)	1.29 (1.22–1.36)
**Combination of parental educational attainment^c^**					
HH (both post-secondary)	32753	6192 (18.9)	1.00 (Reference)	1.00 (Reference)	1.00 (Reference)
HL (father post-secondary and mother secondary)	7798	1847 (23.7)	1.33 (1.26–1.41)	1.24 (1.16–1.31)	1.20 (1.12–1.27)
LH (father secondary and mother post-secondary)	13838	3303 (23.9)	1.35 (1.28–1.41)	1.21 (1.16–1.28)	1.18 (1.12–1.24)
LL (both secondary)	13923	4348 (31.2)	1.95 (1.86–2.04)	1.60 (1.52–1.68)	1.52 (1.44–1.59)

^a^Adjusted for sex, birth weight, maternal age at birth, marital status, mother’s working hours, daycare attendance, siblings, co-residence with grandparents, household smoking, and residential area.

^b^Further adjusted for other socioeconomic variables: model for household income adjusted for paternal and maternal education; models for paternal/maternal education adjusted for equivalized household income and the other parent’s education; combination of parental education model adjusted for equivalized household income.

^c^H (post-secondary education) refers to vocational school, junior college, university, or higher. L (secondary education) refers to high school or less.

Abbreviations: OR, odds ratio; CI, confidence interval.

The associations between parental socioeconomic status and early childhood caries were consistent in the population for the secondary analysis, which excluded children who had caries between birth and 2 years of age (Supplementary Table 2).

### Oral health behaviors and their mediating effects

Tables 3a and 3b describe the distribution of oral health behaviors at age 2 and dental caries between ages 3 and 4 years, respectively, according to household income and parental educational attainment. Overall, approximately 55% of children received fluoride between the ages of 0 and 2 years. Approximately 47% of the children brushed their teeth twice a day or more, and over 95% received parental assistance with the final tooth brushing every time. Approximately 5% of the children continued to be bottle-fed at age 2 years, and approximately 9% received snacks between meals more than three times a day.

**Table 3a:** Oral health behaviors at age 2 and caries at ages 3–4 by household income.

**Oral health behaviors at age 2 years**	**Equivalized household income**							

	**Lower (<2.78 million yen)**				**Higher (≥2.78 million yen)**			
	
	**Participants**		**Case**		**Participants**		**Case**	
			
	**Number (%)**		**Number (%)**		**Number (%)**		**Number (%)**	
Fluoride application during 0–2 years								
Yes	16656	(54.3)	2913	(51.7)	18024	(56.2)	2251	(53.5)
No	13071	(42.6)	2497	(44.3)	13311	(41.5)	1827	(43.4)
Missing	936	(3.1)	226	(4.0)	737	(2.3)	132	(3.1)
Tooth brushing frequency per day								
Twice or more	14588	(47.6)	2504	(44.4)	14734	(45.9)	1689	(40.1)
Once or less	15148	(49.4)	2905	(51.5)	16629	(51.9)	2394	(56.9)
Missing	927	(3.0)	227	(4.0)	709	(2.2)	127	(3.0)
Final tooth brushing								
Always	29194	(95.2)	5289	(93.8)	30920	(96.4)	3990	(94.8)
Sometimes or never	525	(1.7)	121	(2.2)	432	(1.4)	91	(2.2)
Missing	944	(3.1)	226	(4.0)	720	(2.2)	129	(3.1)
Toothpaste use								
Yes	17101	(55.8)	2836	(50.3)	17697	(55.2)	2118	(50.3)
No	12594	(41.1)	2567	(45.6)	13620	(42.5)	1960	(46.6)
Missing	968	(3.2)	233	(4.1)	755	(2.4)	132	(3.1)
Continued bottle feeding								
No	28089	(91.6)	5048	(89.6)	29968	(93.4)	3257	(77.4)
Yes	1643	(5.4)	367	(6.5)	1382	(4.3)	603	(14.3)
Missing	931	(3.0)	221	(3.9)	722	(2.3)	350	(8.3)
Between-meal snacks per day								
Twice or less	24878	(81.1)	4327	(76.8)	26597	(82.9)	3827	(90.9)
3 times or more	3013	(9.8)	762	(13.5)	2966	(9.3)	251	(6.0)
Missing	2772	(9.0)	547	(9.7)	2509	(7.8)	132	(3.1)

**Table 3b:** Oral health behaviors at age 2 and caries at ages 3–4 by parental education.

**Oral health behaviors at age 2 years**	**Parental education**							

	**Both secondary**				**At least one post-secondary**			
	
	**Participants**		**Case**		**Participants**		**Case**	
			
	**Number (%)**		**Number (%)**		**Number (%)**		**Number (%)**	
Fluoride application during 0–2 years								
Yes	6637	(53.5)	1427	(51.8)	28043	(55.7)	3737	(52.7)
No	5262	(42.4)	1186	(43.1)	21120	(42.0)	3138	(44.3)
Missing	509	(4.1)	142	(5.2)	1164	(2.3)	216	(3.1)
Tooth brushing frequency per day								
Twice or more	5914	(47.7)	1238	(44.9)	23408	(46.5)	2955	(41.7)
Once or less	5996	(48.3)	1378	(50.0)	25781	(51.2)	3921	(55.3)
Missing	498	(4.0)	139	(5.1)	1138	(2.3)	215	(3.0)
Final tooth brushing								
Always	11630	(93.7)	2545	(92.4)	48484	(96.3)	6734	(95.0)
Sometimes or never	275	(2.2)	71	(2.6)	682	(1.4)	141	(2.0)
Missing	503	(4.1)	139	(5.1)	1161	(2.3)	216	(3.1)
Toothpaste use								
Yes	7034	(56.7)	1409	(51.1)	27764	(55.2)	3545	(50.0)
No	4860	(39.2)	1203	(43.7)	21354	(42.4)	3324	(46.9)
Missing	514	(4.1)	143	(5.2)	1209	(2.4)	222	(3.1)
Continued bottle feeding								
No	11122	(89.6)	2411	(87.5)	46935	(93.3)	5549	(78.3)
Yes	782	(6.3)	205	(7.4)	2243	(4.5)	965	(13.6)
Missing	504	(4.1)	139	(5.1)	1149	(2.3)	577	(8.1)
Between-meal snacks per day								
Twice or less	9717	(78.3)	2035	(73.9)	41758	(83.0)	6464	(91.2)
3 times or more	1372	(11.1)	400	(14.5)	4607	(9.2)	413	(5.8)
Missing	1319	(10.6)	320	(11.6)	3962	(7.9)	214	(3.0)

Post-secondary education refers to vocational school, junior college, university, or higher education. Secondary education refers to high school or lower.

Both household income and parental educational attainment were positively associated with preventive oral health behaviors, particularly fluoride application, final tooth brushing, and discontinued bottle feeding in this cohort (Table 4).

**Table 4:** Odds ratios and 95% confidence intervals of each oral health behavior according to socioeconomic factors.

**Socioeconomic factors**	**Fluoride** **Adjusted^a^ OR** **(95% CI)**	**Brushing frequency** **Adjusted^a^ OR** **(95% CI)**	**Final brushing** **Adjusted^a^ OR** **(95% CI)**	**Toothpaste** **Adjusted^a^ OR** **(95% CI)**	**Bottle feeding** **Adjusted^a^ OR** **(95% CI)**	**Snacks** **Adjusted^a^ OR** **(95% CI)**
**Equivalized household income**						
Higher (≥2.78 million yen)	1.00 (Reference)	1.00 (Reference)	1.00 (Reference)	1.00 (Reference)	1.00 (Reference)	1.00 (Reference)
Lower (<2.78 million yen)	0.85 (0.81–0.88)	1.01 (0.97–1.05)	0.83 (0.72–0.97)	0.93 (0.90–0.96)	0.86 (0.79–0.94)	0.99 (0.94–1.06)
**Combination of parental educational attainment^b^**						
At least one post-secondary	1.00 (Reference)	1.00 (Reference)	1.00 (Reference)	1.00 (Reference)	1.00 (Reference)	1.00 (Reference)
Both secondary	0.92 (0.88–0.67)	1.03 (0.99–1.08)	0.60 (0.52–0.71)	0.99 (0.95–1.04)	0.75 (0.68–0.82)	0.84 (0.78–0.90)

Definition of oral health behaviors: fluoride application (yes vs. no [reference]); brushing frequency (≥2 times/day vs. <2 times/day [reference]); final tooth brushing (always vs. not always [reference]); toothpaste use (yes vs. no [reference]); continued bottle feeding (yes vs. no [reference]); between-meal snacks (≤2 times/day vs. ≥3 times/day [reference]).

^a^Adjusted for sex, birth weight, maternal age at birth, marital status, mother’s working hours, daycare attendance, siblings, co-residence with grandparents, household smoking, residential area, and the other socioeconomic variables: model for equivalized household income adjusted for combination of parental education model; and model for combination of parental education model adjusted for equivalized household income.

^b^Post-secondary education refers to vocational school, junior college, university, or higher education. Secondary education refers to high school or lower.

Abbreviations: OR, odds ratio; CI, confidence interval.

Fluoride application, final tooth brushing, toothpaste use, and continued bottle feeding mediated the association between household income and caries (Table 5a). Conversely, fluoride application, final tooth brushing, continued bottle feeding, and the frequency of between-meal snacks mediated the association between parental educational attainment and dental caries (Table 5b). However, the estimated mediating effect of each oral health behavior was small, accounting for less than 5% of the total effect.

**Table 5a:** Estimated effects of household income on dental caries mediated by oral health behaviors.

**Oral health behaviors**	**Adjusted Odds Ratio^a^**	**(95% CI^b^)**
Fluoride application (Reference: yes)		
Total effect	1.18	(1.12, 1.24)
Natural direct effect	1.18	(1.11, 1.24)
Natural indirect effect	1.01	(1.004, 1.01)
Percentage mediated (%)	3.86 (%)	(2.29%, 6.70%)
Brushing frequency (Reference: twice or more per day)		
Total effect	1.18	(1.12, 1.24)
Natural direct effect	1.18	(1.13, 1.25)
Natural indirect effect	1.00	(0.997, 1.002)
Percentage mediated (%)	−0.34 (%)	(−1.93%, 0.95%)
Final tooth brushing (Reference: always)		
Total effect	1.18	(1.12, 1.24)
Natural direct effect	1.18	(1.12, 1.24)
Natural indirect effect	1.00	(1.000, 1.002)
Percentage mediated (%)	0.65 (%)	(0.14%, 1.61%)
Toothpaste use (Reference: yes)		
Total effect	1.18	(1.13, 1.24)
Natural direct effect	1.18	(1.12, 1.24)
Natural indirect effect	1.01	(1.003, 1.01)
Percentage mediated (%)	3.51 (%)	(1.75%, 6.05%)
Continued bottle feeding (Reference: no)		
Total effect	1.18	(1.12, 1.24)
Natural direct effect	1.18	(1.12, 1.24)
Natural indirect effect	1.00	(1.001, 1.003)
Percentage mediated (%)	1.18 (%)	(0.49%, 2.32%)
Between-meal snacks (Reference: twice or less per day)		
Total effect	1.18	(1.12, 1.24)
Natural direct effect	1.18	(1.12, 1.24)
Natural indirect effect	1.00	(0.998, 1.003)
Percentage mediated (%)	0.20 (%)	(−1.92%, 2.33%)

^a^Adjusted for sex, birth weight, maternal age at birth, marital status, mother’s working hours, daycare attendance, siblings, co-residence with grandparents, household smoking, residential area, and parental educational attainment.

^b^Boot strap 95% CI

Reference category of equivalized household income was higher (≥2.78 million yen) group. All oral health behaviors were measured at age 2.

Abbreviations: CI, confidence interval.

**Table 5b:** Estimated effects of parental education on dental caries mediated by oral health behaviors.

**Oral health behaviors**	**Adjusted Odds Ratio^a^**	**(95% CI^b^)**
Fluoride application (Reference: yes)		
Total effect	1.41	(1.33, 1.49)
Natural direct effect	1.41	(1.32, 1.49)
Natural indirect effect	1.00	(1.001, 1.01)
Percentage mediated (%)	1.03 (%)	(0.46%, 1.98%)
Brushing frequency (Reference: twice or more per day)		
Total effect	1.41	(1.33, 1.49)
Natural direct effect	1.41	(1.33, 1.49)
Natural indirect effect	0.998	(0.996, 1.001)
Percentage mediated (%)	−0.60 (%)	(−1.59%, 0.34%)
Final tooth brushing (Reference: always)		
Total effect	1.41	(1.34, 1.50)
Natural direct effect	1.40	(1.34, 1.49)
Natural indirect effect	1.003	(1.002, 1.006)
Percentage mediated (%)	1.12 (%)	(0.56%, 2.02%)
Toothpaste use (Reference: yes)		
Total effect	1.41	(1.32, 1.49)
Natural direct effect	1.41	(1.33, 1.49)
Natural indirect effect	1.001	(0.998, 1.004)
Percentage mediated (%)	0.18 (%)	(−0.82%, 1.42%)
Continued bottle feeding (Reference: no)		
Total effect	1.41	(1.34, 1.50)
Natural direct effect	1.40	(1.34, 1.49)
Natural indirect effect	1.004	(1.002, 1.007)
Percentage mediated (%)	1.35 (%)	(0.74%, 2.17%)
Between-meal snacks (Reference: twice or less per day)		
Total effect	1.41	(1.33, 1.49)
Natural direct effect	1.40	(1.31, 1.47)
Natural indirect effect	1.01	(1.01, 1.01)
Percentage mediated (%)	2.75 (%)	(1.65%, 4.33%)

^a^Adjusted for sex, birth weight, maternal age at birth, marital status, mother’s working hours, daycare attendance, siblings, co-residence with grandparents, household smoking, residential area, and equivalized household income.

^b^Boot strap 95% CI

The reference category for parental education was at least one parent with post-secondary education. All oral health behaviors were measured at age two.

Abbreviations: CI, confidence interval.

## Discussion

In this nationwide prospective cohort study of Japanese children, we found that disparities in the prevalence of dental caries according to parental SES emerged at age 2. The gap in the cumulative prevalence of caries progressively widened with both decreasing household income and lower parental education. In addition to the cumulative prevalence of having at least one caries diagnosis, a socioeconomic gradient was also observed when classifying children by the number of years with diagnosis. After adjusting for potential confounders, children from lower-income households and those with at least one parent with secondary education had higher odds of developing dental caries. Notably, children whose parents had attained secondary education had 56% higher odds than those whose parents had attained post-secondary education. Fluoride application, final tooth brushing, toothpaste use, continued bottle feeding, and the frequency of between-meal snacks mediated the association between SES and caries. However, the estimated mediating effects of each behavior were minor, each accounting for less than 5% of the total effect.

Several studies in Japan have examined the association between SES and dental caries in preschoolers. A study of 315 children aged 41–50 months reported that higher parental educational attainment, particularly maternal education, was associated with lower odds of caries, whereas household income was not [16]. The adjusted OR (95% CI) of dental caries for maternal education ≥15 years compared with <13 years was 0.32 (0.14–0.70), while the corresponding OR for paternal education ≥15 years was 0.45 (0.23–0.87). Another study of approximately 6,300 children aged 36–47 months found that higher household income and higher maternal and paternal educational attainment were independently associated with lower odds of caries [17]. The adjusted ORs of dental caries for maternal and paternal education of ≥14 years compared to <13 years were 0.64 (0.53–0.77) and 0.69 (0.59–0.82), respectively. In our study, both maternal and paternal education were independently associated with dental caries. There appeared to be little difference in the strength of association of maternal and paternal education with dental caries; the adjusted ORs for high school education or less (vs. university or higher) were 1.33 (1.25–1.40) for mothers and 1.26 (1.20–1.32) for fathers. Notably, none of the previous studies considered the combined effects of paternal and maternal educational attainment. Extending prior work, we examined combined classifications, which showed a stronger association than when assessing each parent’s education separately.

Another nationwide study utilizing a large cohort of Japanese children born in 2001, which used the same combined classification of paternal and maternal education as in the present study, reported that disparities in the proportion of caries treatment were already evident at age 2.5 years and continued to widen through 5.5 years [8]. Our findings are consistent with this result, showing that disparities in the prevalence of caries by parental SES began to emerge at age 2 and became more pronounced as the children grew older. These results, based on two distinct nationwide birth cohorts conducted roughly a decade apart, indicate that socioeconomic disparities in early ECC persist in Japan.

Parents play a key role in maintaining children’s oral health [6]. Maternal education has consistently been associated with a lower prevalence of ECC, whereas its association with paternal education has been variable across studies [18]. Feeding practices, oral hygiene habits, and the use of dental services may mediate the association between maternal education and ECC [18]. Although maternal education has shown a stronger association with ECC than paternal education, both have been independently associated with ECC in previous Japanese studies [16, 17]. In our study, a strong gradient in the odds of caries was observed based on the combination of parental education: the odds were lowest when both parents had completed post-secondary education, intermediate when only one parent had attained secondary education, and highest when neither parent had completed secondary education. Among the discordant pairs, little difference was observed in whether the mother or father had a higher education. The elevated odds among children whose parents had attained secondary-level education may reflect the absence of protective factors that are more likely to be present when at least one parent has attained higher education. For example, higher parental education may be associated with greater awareness of oral health recommendations and more consistent implementation of preventive behaviors such as regular tooth brushing and appropriate use of fluoride [19, 20]. However, the observed associations of household income and parental education with early childhood caries do not necessarily imply that these effects operate solely through access to preventive dental items or parental knowledge. Particularly in the Japanese context, where basic preventive resources are relatively accessible, socioeconomic gradients in childhood caries may reflect a broader set of social and behavioral factors. The extent of parental attention to children’s diet may also influence the quality of their diet, including the frequency and type of between-meal snacks [21]. In families in which both parents had attained secondary education, such behaviors may be less frequently demonstrated by parents or encouraged in daily routines. Furthermore, parents with higher education may be more likely to compensate for gaps in their partners’ practices [22]. Additionally, health literacy may influence the ability to interpret dental health information or navigate preventive services [23], potentially affecting children’s oral health routines and access to care. Although universal dental checkup programs are available free of charge nationwide at 18 months and 3 years of age, and attendance rates consistently exceed 90% [24], there are concerns about the small proportion of children who do not attend these programs, as they may be at increased risk of developing oral health problems later in life.

We conducted a mediation analysis to evaluate the extent to which oral health behaviors explain the association between SES and ECC. The results indicated that oral health behaviors, including fluoride application, final tooth brushing, toothpaste use, continued bottle feeding, and the frequency of between-meal snacks, partially mediated the association between SES and ECC. Although each of these behaviors has been identified in previous studies as important for preventing ECC [25], their contribution to explaining socioeconomic disparities was small in our analysis, accounting for less than 5% of the total effect. These findings suggest that socioeconomic disparities in early childhood caries cannot be attributed solely to specific parental practices, and that early childhood caries reflects a multifactorial aspect rather than the effect of any single behavior.

The small mediating effects observed for individual oral health behaviors may highlight the need to consider a broader set of determinants, including not only behavioral or individual factors but also upstream factors such as community characteristics, national and local policies, and regional differences on dental care utilization [26, 27]. For instance, many municipalities have offered fluoride application programs free of charge or at low cost for preschool children [28], and approximately half of the children received them by age 2 in the present study. While a population approach aims to improve overall public health, it may not sufficiently address the needs of socioeconomically disadvantaged populations. Taking the social gradient into account may enhance the effectiveness of population-wide strategies in reducing health inequalities. Meanwhile, in later childhood, school-based fluoride mouth-rinse programs have been shown to effectively mitigate socioeconomic disparities in dental caries among children aged 12 years in Japan [29]. Further research focusing on upstream social determinants may contribute to promoting lifelong oral health.

The use of a large nationwide cohort of children born in the 2010s provides updated findings that are broadly reflective of the Japanese population. In addition, the prospective design of the cohort enabled the observation of dental caries development from birth to age 4, allowing for the assessment of when and how socioeconomic disparities in oral health begin to emerge and evolve. Additionally, the longitudinal data ensured a clear temporal sequence, enabling the assessment of baseline SES, oral health practices at 2 years of age, and dental caries outcomes at 3–4 years of age.

However, several limitations should be acknowledged. First, all information on dental caries was obtained from a self-reported questionnaire completed by the guardian rather than from hospital or health checkup records. The questionnaire asked whether the child had been diagnosed with dental caries at each age from 0–4 years, but did not capture the number of affected teeth or the frequency of diagnoses within a year. Therefore, the data indicate only the presence or absence of dental caries each year and not the severity or recurrence. Additionally, this information was collected retrospectively at age 4, relying on guardians’ recall of past diagnoses, which may have introduced recall bias and limited the accuracy of age-specific data. Nevertheless, it is possible that some guardians referred to the Japanese Maternal and Child Health Handbook when completing the questionnaire, as it contains designated sections for recording the results of health checkups at 18 months and 3 years of age [28, 30]. In this case, the extent of the recall error may have been partially mitigated. The age-specific prevalence observed in our study is broadly consistent with national estimates. According to Japan’s National Dental Examination for children aged 18 months and 3 years, the prevalence of caries was approximately 1–2% at 18 months and 12–18% at 3 years during the period from 2014–2018 [31]. In our study, the prevalence was approximately 2% at age 1, 7% at age 2, and 17% at age 3, indicating similar trends across ages. Second, the information on paternal educational attainment was obtained from self-report questionnaires completed by mothers and was not directly reported by fathers. Therefore, some degree of misclassification of paternal education cannot be excluded. Third, household income was categorized using uniform thresholds that did not account for regional differences in the cost of living across Japan. Thus, households with similar nominal incomes may have had different levels of economic resources depending on their region of residence. Fourth, oral health behaviors used as mediators were collected using the age 2 questionnaires and may be subject to misclassification. In particular, fluoride application was assessed as a binary indicator of whether the child had ever received fluoride application between birth and age 2, without information on timing or frequency. In addition, oral health behaviors were dichotomized for analytical feasibility in the mediation analysis, which may have led to loss of information and warrants careful interpretation. Fifth, information on the primary caregiver for children’s oral health care and their educational attainment was not available in this study; therefore, its potential influence on children’s oral health behaviors could not be examined. Sixth, the possibility of selection bias due to missing data cannot be ruled out. Approximately 30% of the participants were excluded from the primary analyses because of incomplete data on key variables, dental caries, or socioeconomic exposures. Systematic patterns in missing data may have led to biased estimates and reduced the generalizability of the results.

## Conclusion

This nationwide prospective cohort study showed that socioeconomic disparities in dental caries among Japanese children emerged as early as age 2 and progressively widened by age 4. Lower household income and parental educational attainment were independently associated with higher odds of dental caries, with particularly high risk observed among children whose parents had secondary-level education. Early oral health behaviors, such as fluoride application and tooth brushing practices, accounted for a small part of socioeconomic inequalities in ECC.

## Abbreviations

ECC: Early childhood caries
SES: Socioeconomic status
JECS: Japan environment and children’s study
OR: Odds ratio
CI: Confidence interval

## Supplementary information

**Additional file 1:** **Supplemental Table 1.** Odds ratios and 95% confidence intervals of early childhood caries according to socioeconomic factors restricted to children with married mothers (N = 66,009). **Supplemental Table 2.** Odds ratios and 95% confidence intervals of early childhood caries occurred between ages 3 and 4 years according to socioeconomic factors for the secondary analysis (N = 62,735). **Supplemental Figure 1.** Flow chart of the participant selection.
