# Supplementary material for: Associations of household income and parental education with early childhood caries: the Japan Environment and Children’s Study
Source: Environ Health Prev Med. 2026 Mar 26;31:23. doi: 10.1265/ehpm.25-00358 (PMC13057869; doi:10.1265/ehpm.25-00358)
Supplement: Supplementary file 1 — Additional file 1: Supplemental Table 1. Odds ratios and 95% confidence intervals of early childhood caries according to socioeconomic factors restricted to children with married mothers (N = 66,009). Supplemental Table 2. Odds ratios and 95% confidence intervals of early childhood caries occurred between ages 3 and 4 years according to socioeconomic factors for the secondary analysis (N = 62,735). Supplemental Figure 1. Flow chart of the participant selection. [file ehpm-31-023-s001.docx]

**Supplemental Table 1. Odds ratios and 95% confidence intervals of early childhood caries according to socioeconomic factors restricted to children with married mothers** **(N = 66,009).**

| Socioeconomic factors | | No. of  participants | No. (%)  of case | | Crude OR (95% CI) | | Adjusted OR^a^  (95% CI) | |
| --- | --- | --- | --- | --- | --- | --- | --- | --- |
| Equivalized household income | |  |  |  |  |  |  |  |
|  | Q4, highest (≥ 3.33 million yen) | 15622 | 2792 | (17.9) | 1.00 | (Reference) | 1.00 | (Reference) |
|  | Q3 (2.78 –< 3.33 million yen) | 18141 | 3682 | (20.3) | 1.17 | (1.11–1.24) | 1.05 | (1.00–1.11) |
|  | Q2 (1.67 –< 2.78 million yen) | 14840 | 3458 | (23.3) | 1.40 | (1.32–1.48) | 1.17 | (1.10–1.25) |
|  | Q1, lowest (< 1.67 million yen) | 17406 | 5122 | (29.4) | 1.92 | (1.82–2.02) | 1.25 | (1.17–1.33) |
| Paternal educational attainment | |  |  |  |  |  |  |  |
|  | University or higher | 24404 | 4482 | (18.4) | 1.00 | (Reference) | 1.00 | (Reference) |
|  | Vocational school or junior college | 15272 | 3353 | (22.0) | 1.25 | (1.19–1.32) | 1.08 | (1.02–1.14) |
|  | High school or less | 26333 | 7219 | (27.4) | 1.68 | (1.61–1.75) | 1.25 | (1.19–1.31) |
| Maternal educational attainment | |  |  |  |  |  |  |  |
|  | University or higher | 16559 | 2958 | (17.9) | 1.00 | (Reference) | 1.00 | (Reference) |
|  | Vocational school or junior college | 28988 | 6287 | (21.7) | 1.27 | (1.21–1.34) | 1.06 | (1.01–1.12) |
|  | High school or less | 20462 | 5809 | (28.4) | 1.82 | (1.73–1.92) | 1.30 | (1.23–1.38) |
| Combination of parental educational attainment^b^ | | |  |  |  |  |  |  |
|  | HH (both post-secondary) | 32196 | 6070 | (18.9) | 1.00 | (Reference) | 1.00 | (Reference) |
|  | HL (father post-secondary and mother secondary) | 7480 | 1765 | (23.6) | 1.33 | (1.25–1.41) | 1.20 | (1.13–1.28) |
|  | LH (father secondary and mother post-secondary) | 13351 | 3175 | (23.8) | 1.34 | (1.28–1.41) | 1.18 | (1.12–1.24) |
|  | LL (both secondary) | 12982 | 4044 | (31.2) | 1.95 | (1.86–2.04) | 1.53 | (1.45–1.61) |

^a^Adjusted for sex, birth weight, maternal age at birth, mother’s working hours, daycare attendance, siblings, co-residence with grandparents, household smoking, residential area, and other socioeconomic variables (model for equivalized household income adjusted for paternal and maternal education; models for paternal/maternal education adjusted for equivalized household income and the other parent's education; combination of parental education model adjusted for equivalized household income).

^b^H (post-secondary education) refers to vocational school, junior college, university, or higher. L (secondary education) refers to high school or less.

Abbreviations: OR, odds ratio; CI, confidence interval.

**Supplemental Table 2. Odds ratios and 95% confidence intervals of early childhood caries occurred between ages 3 and 4 years according to socioeconomic factors for the secondary analysis** **(N = 62,735).**

| Socioeconomic factors | | No. of  participants | No. (%)  of case | | Crude OR (95% CI) | | Adjusted OR^a^  (95% CI) | |
| --- | --- | --- | --- | --- | --- | --- | --- | --- |
| Equivalized household income | |  |  |  |  |  |  |  |
|  | Q4, highest (≥ 3.33 million yen) | 14921 | 1800 | (12.1) | 1.00 | (Reference) | 1.00 | (Reference) |
|  | Q3 (2.78 –< 3.33 million yen) | 17151 | 2410 | (14.1) | 1.19 | (1.12–1.27) | 1.07 | (1.00–1.15) |
|  | Q2 (1.67 –< 2.78 million yen) | 14165 | 2239 | (13.8) | 1.38 | (1.29–1.47) | 1.14 | (1.06–1.23) |
|  | Q1, lowest (< 1.67 million yen) | 16498 | 3397 | (20.6) | 1.92 | (1.80–2.04) | 1.23 | (1.15–1.32) |
| Paternal educational attainment | |  |  |  |  |  |  |  |
|  | University or higher | 23160 | 2813 | (12.2) | 1.00 | (Reference) | 1.00 | (Reference) |
|  | Vocational school or junior college | 14526 | 2222 | (15.3) | 1.32 | (1.24–1.40) | 1.12 | (1.05–1.19) |
|  | High school or less | 25049 | 4811 | (19.2) | 1.73 | (1.64–1.82) | 1.26 | (1.05–1.32) |
| Maternal educational attainment | |  |  |  |  |  |  |  |
|  | University or higher | 15747 | 1879 | (11.9) | 1.00 | (Reference) | 1.00 | (Reference) |
|  | Vocational school or junior college | 27439 | 4057 | (14.8) | 1.29 | (1.21–1.36) | 1.06 | (0.99–1.12) |
|  | High school or less | 19549 | 3910 | (20.0) | 1.86 | (1.75–1.97) | 1.30 | (1.22–1.40) |
| Combination of parental educational attainment^b^ | | |  |  |  |  |  |  |
|  | HH (both post-secondary) | 30545 | 3880 | (12.7) | 1.00 | (Reference) | 1.00 | (Reference) |
|  | HL (father post-secondary and mother secondary) | 7141 | 1155 | (16.2) | 1.33 | (1.24–1.43) | 1.19 | (1.11–1.28) |
|  | LH (father secondary and mother post-secondary) | 12641 | 2056 | (16.3) | 1.33 | (1.26–1.41) | 1.16 | (1.09–1.23) |
|  | LL (both secondary) | 12408 | 2755 | (15.7) | 1.97 | (1.87–2.10) | 1.51 | (1.43–1.61) |

^a^Adjusted for sex, birth weight, maternal age at birth, marital status, mother’s working hours, daycare attendance, siblings, co-residence with grandparents, household smoking, residential area, and other socioeconomic variables (model for equivalized household income adjusted for paternal and maternal education; models for paternal/maternal education adjusted for equivalized household income and the other parent's education; combination of parental education model adjusted for equivalized household income).

^b^H (post-secondary education) refers to vocational school, junior college, university, or higher. L (secondary education) refers to high school or less.

Abbreviations: OR, odds ratio; CI, confidence interval.

**Supplemental Figure 1. Flow chart of the participant selection**

| All fetal records | |  |  |
| --- | --- | --- | --- |
| n = 104,043 | |  |  |
|  |  |  | Excluded owing to stillbirths, miscarriages, or abortions (n = 3,921) |
|  |  |  |  |
| Live births | |  |  |
| n = 100,122 | |  |  |
|  |  |  | Excluded owing to non-response to age 4 questionnaire (n = 22,075) |
|  |  |  |  |
|  |  |  |  |
|  |  |  | Excluded owing to missing information on caries diagnoses (n = 3,826) |
|  |  |  |  |
|  |  |  |  |
|  |  |  | Excluded owing to missing information on household income and/or |
|  |  |  | parental educational attainment (n = 5,467) |
|  |  |  |  |
|  |  |  | Excluded owing to missing information on the number of household members |
|  |  |  | (n = 442) |
|  |  |  |  |
| Primary analysis | |  |  |
| n = 68,312 | |  |  |
|  |  |  | Excluded owing to caries diagnoses before age 2 (n = 5,577) |
|  |  |  |  |
| Secondary analysis | |  |  |
| n = 62,735 | |  |  |
